# Administration of combined venetoclax and azacitidine in a patient with acute myeloid leukemia and multiple comorbidities undergoing dialysis: A case report

**DOI:** 10.1002/jha2.732

**Published:** 2023-06-06

**Authors:** Yuina Akagi, Yusuke Yamashita, Hideki Kosako, Yoshiaki Furuya, Hiroki Hosoi, Toshiki Mushino, Shogo Murata, Akinori Nishikawa, Shinobu Tamura, Taisei Nakao, Takashi Sonoki

**Affiliations:** ^1^ Department of Hematology/Oncology Wakayama Medical University Wakayama Japan; ^2^ Department of Hematology Naga Municipal Hospital Wakayama Japan; ^3^ Department of Internal Medicine Naga Municipal Hospital Wakayama Japan

**Keywords:** acute myeloid leukemia, azacitidine, dialysis, venetoclax

## Abstract

Patients with acute myeloid leukemia (AML) who have comorbidities have limited treatment options, thereby resulting in poor prognosis. Venetoclax, a specific B‐cell lymphoma‐2 inhibitor, has recently been approved for AML in combination with hypomethylating agents; however, only one report has described its use in patients undergoing dialysis. Herein, we report the effectiveness of combined venetoclax and azacitidine in a 73‐year‐old man with AML undergoing dialysis and who was ineligible for standard therapies. The safety of venetoclax and azacitidine in patients undergoing dialysis has been reported, and their combination may be a feasible option for patients with AML undergoing dialysis.

## 1 INTRODUCTION

AML is a hematopoietic tumor characterized by abnormal proliferation and differentiation arrests of myeloid progenitor cells [1]. Standard therapies are intensive chemotherapies with highly toxic, thus elderly patients and those with comorbidities were ineligible for these therapies. Therefore, their treatment options are limited and prognosis is very poor, especially in dialysis patients [1, 2]. Recently, venetoclax, a B‐cell lymphoma‐2 (BCL‐2) inhibitors, can be used in elderly and frail patients, greatly improving their prognosis [2, 3, 4]. Herein, we report the effective administration of combined venetoclax and azacitidine in an elderly AML patient who was ineligible for standard therapies undergoing dialysis. To the best of our knowledge, there is only one report regarding the use of combined venetoclax and azacitidine in dialysis patient. He was eligible for standard chemotherapy, and the combination was used at relapse [5]. Therefore, our case is the first report of the use of combination therapy in a dialysis patient who was ineligible for standard therapy.

## 2 CASE REPORT

A 73‐year‐old Japanese man receiving hemodialysis was referred to our hospital with pancytopenia. He had a medical history of distal gastrectomy, right putaminal hemorrhage (no sequelae), and asymptomatic myocardial ischemia (first‐degree atrioventricular block). His pancytopenia was asymptomatic and detected via regular blood tests. Bone marrow aspiration (BMA) showed the increased blasts (nuclear cell count 35,000/μL and blasts count 7.8%), but no dysplasia in the three lineages. Moreover, flow cytometry revealed that increased blasts expressed CD13, CD33, CD34, and HLA‐DR. *CBFβ‐MYH11* mRNA (1.4 × 10^4^ copies/μgRNA) was detected, and cytogenetic analysis revealed 46, XY, del [7] (q?), inv(16)(p13;1q22) in eight of 20 metaphase cells. Therefore, the final diagnosis was acute myeloid leukemia with inv(16)(p13;1q22) or t(16;16)(p13.1;q22); *CBFB‐MYH11*.

**FIGURE 1:**
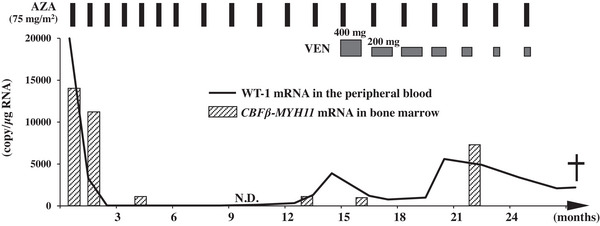
Patient's clinical course. Overall, 12 cycles of AZA monotherapy (AZA: 75 mg/m^2^/day on days 1–7, 4–6‐week‐cycle) and 7 cycles of combination therapy (VEN: 400 mg/day on days 1–28 tapered to 200 mg/day on days 1–28, 200 mg/day on days 1–14, and 200 mg/day on days 1–10. AZA: 75 mg/m^2^/day on days 1–7, 6‐week‐cycle) were administered. AZA, azacitidine; VEN, venetoclax. N.D., not detected (*CBFβ‐MYH11* mRNA in bone marrow).

After diagnosis, azacitidine (AZA) treatment was initiated upon hospitalization at the standard dose (75 mg/m^2^/day subcutaneously for 7 days every 4 weeks). The subsequent clinical course is shown in (Figure 1). After the second cycle, he developed febrile neutropenia and required treatment with cefepime. There were no other significant adverse events, and his blasts in the peripheral blood disappeared. After the third cycle, the white blood cell and platelet counts normalized, and the red blood cell transfusion was no longer necessary. Consequently, he was discharged. Thereafter, he was hospitalized only for AZA administration but was treated as an outpatient. BMA after the fourth cycle showed hematological complete remission. Owing to grade 4 neutropenia, the treatment cycle was changed to a 6‐week cycle from the seventh cycle, but he was able to continue AZA treatment without major adverse events other than neutropenia.

However, 15 months after treatment initiation, levels of WT‐1 mRNA levels showed an upward trend, although regular BMA showed persistent hematological remission. He was diagnosed with molecular relapse and started combination therapy of venetoclax and AZA (venetoclax 400 mg/day orally for days 1–28, AZA 75 mg/m^2^/day subcutaneously for days 1–7, 6‐week‐cycle) without antibiotic and antifungal prophylaxis. Although WT‐1 mRNA levels decreased, dose reduction of venetoclax was necessary (stepwise change to 200 mg/day on days 1–10). Other than neutropenia, no major adverse events occurred. However, 12 months after combination therapy initiation, the patient was hospitalized due to fever despite a normal white blood cell count. Empiric therapy with meropenem was administered, but he eventually died due to sepsis caused by *Escherichia coli*. Hematological remission was maintained, and no signs of AML relapse were observed, suggesting that AML was controlled by combination therapy.

## 3 DISCUSSION

Effective treatment of AML is challenging in elderly patients or those with comorbidities, particularly in dialysis patients [2]. Venetoclax combined with azacitidine or cytarabine has favorable therapeutic effects for the AML patients who are ineligible for intensive chemotherapy [3, 4], therefore, expanding their treatment options. Additionally, there is a report that adding venetoclax as treatment of AML relapse after azacitidine treatment is effective [6]. Combination therapy may be an option not only for initial treatment but also post azacitidine therapy.

Venetoclax is a highly selective BCL‐2 inhibitor and is primarily eliminated via hepatic enzymatic activity [7], so its administration in patients with renal failure theoretically be safe. Indeed, venetoclax was reportedly safe for dialysis‐dependent patients with multiple myeloma [8]. Recently, venetoclax was reportedly administered in patients with AML undergoing dialysis eligible for standard therapies [5]. Hence, venetoclax is expected to be safe in dialysis patients.

AZA is a DNA methylation inhibitor that undergoes spontaneous hydrolysis in aqueous solutions and is rapidly deaminated and subsequent degradation [9]. Most metabolites/degradation products of AZA are excreted in urine [10]. In dialysis patients, there is concern not only about toxicity due to drug accumulation but also about decreased efficacy due to drug removal, because AZA is removed by hemodialysis [11]. However, most of the reported patients have been administered with standard doses, which resulted in sufficient efficacy without serious adverse events [11, 12, 13].

In the present case, complete remission was maintained with AZA monotherapy for approximately 15 months, and subsequent molecular relapse was controlled by adding venetoclax to AZA. Despite dialysis and multiple comorbidities, this patient tolerated the combination therapies, resulting in hematological remission for the 12 months. Elderly AML patients ineligible for standard therapy have a poor prognosis, with a median overall survival of only several months for BSC and 5.0–7.7 months for low‐dose AraC or decitabine [14, 15]. Meanwhile, our patient survived for 27 months with AZA monotherapy followed by combined venetoclax and AZA. AZA monotherapy, followed by the combination therapies, may be an effective and feasible treatment option even in dialysis patients ineligible for standard therapy, although infections must be considered in these patients.

To the best our knowledge, this is the first report of combination therapy with venetoclax and azacitidine in a AML patient receiving dialysis ineligible for standard therapies. This report suggests that the combination therapy may be an effective and feasible treatment option in patients with AML and multiple comorbidities undergoing dialysis. However, accumulation of cases is required to confirm the efficacy and safety of this combination therapy in patients undergoing dialysis.

## AUTHOR CONTRIBUTIONS

YA and YY wrote the manuscript. YA, YY, HK, YF, HH, TM, SM, AN, and ST conducted the clinical diagnosis and treatment. TN and TS supervised the clinical diagnosis and treatment. All authors read and approved the final manuscript.

## CONFLICT OF INTEREST STATEMENT

The author declares they have no conflict of interest.

## ETHICS STATEMENT

Our patient and his family members provided informed consent for the publication of his data and any related information.

## Data Availability

The data that support the findings of this study are available upon request from the corresponding author.
